# The role of heterogeneity in contact timing and duration in network models of influenza spread in schools

**DOI:** 10.1098/rsif.2015.0279

**Published:** 2015-07-06

**Authors:** Damon J. A. Toth, Molly Leecaster, Warren B. P. Pettey, Adi V. Gundlapalli, Hongjiang Gao, Jeanette J. Rainey, Amra Uzicanin, Matthew H. Samore

**Affiliations:** 1 Department of Internal Medicine, University of Utah, Salt Lake City, UT 84132, USA; 2 Department of Mathematics, University of Utah, Salt Lake City, UT 84112, USA; 3 Department of Pathology, University of Utah, Salt Lake City, UT 84112, USA; 4 VA Salt Lake City Health Care System, Salt Lake City, UT 84108, USA; 5 Department of Biomedical Informatics, University of Utah, Salt Lake City, UT 84108, USA; 6 Division of Global Migration and Quarantine, Centers for Disease Control and Prevention, Atlanta, GA 30333, USA

**Keywords:** contact network, epidemiology, influenza, schools, mathematical model

## Abstract

Influenza poses a significant health threat to children, and schools may play a critical role in community outbreaks. Mathematical outbreak models require assumptions about contact rates and patterns among students, but the level of temporal granularity required to produce reliable results is unclear. We collected objective contact data from students aged 5–14 at an elementary school and middle school in the state of Utah, USA, and paired those data with a novel, data-based model of influenza transmission in schools. Our simulations produced within-school transmission averages consistent with published estimates. We compared simulated outbreaks over the full resolution dynamic network with simulations on networks with averaged representations of contact timing and duration. For both schools, averaging the timing of contacts over one or two school days caused average outbreak sizes to increase by 1–8%. Averaging both contact timing and pairwise contact durations caused average outbreak sizes to increase by 10% at the middle school and 72% at the elementary school. Averaging contact durations separately across within-class and between-class contacts reduced the increase for the elementary school to 5%. Thus, the effect of ignoring details about contact timing and duration in school contact networks on outbreak size modelling can vary across different schools.

## 1. Introduction

School-age children bear a significant burden of illness caused by influenza infection [1,2]. High rates of transmission among students may also pose direct risk to family contacts [3] and indirect risk to their larger community [4]. Quantifying transmission-relevant contacts among children at schools is an important component of mathematical transmission models and simulations. These in turn serve to clarify the role that schools play in community transmission and the potential effects of school closures or other school-based interventions in reducing transmission.

Influenza virus can be transmitted via three route categories: (i) large droplets expelled by an infectious person through coughing, sneezing or talking directly into the eyes, nose or mouth of a susceptible person; (ii) smaller, aerosolized particles inhaled by a susceptible person; and (iii) hand-to-face self-inoculation after touching a contaminated person, surface or object [5]. Specific scenarios within each category involve an infectious and susceptible pair being in simultaneous, close proximity. Large droplets travel only short distances, typically 6 feet or less [6]. Aerosolized particles can travel further, but a recent study found that detected amounts of influenza-carrying small particles emitted by infected hospital patients dropped significantly at a distance of 6 feet from the patients, compared with closer distances [7]. Finally, hand contamination via direct touching or object passing requires proximity within arm's reach. Therefore, while the relative importance of transmission modes or particular scenarios remains unclear, contacts within close proximity (approximately 6 feet or 2 metres) are thought to be highly relevant to influenza transmission.

Objective data on close-proximity contact among children or adults have been scarce until recently, as researchers have deployed wearable, wireless sensor networks to people in schools [8–13] and other settings [14–16]. These data provide granular information about close-proximity contacts over time and space, and transmission modellers must decide whether to use complicated models incorporating those details or simpler models parametrized by averages of the data. While some have systematically assessed the relevance of granular contact details to outbreak models for diseases with influenza-like transmission characteristics [15,17], more work is needed in this area [18,19].

In this paper, we present two new contact datasets we collected at two schools in the state of Utah, USA, together with a novel, individual-based mathematical transmission model for influenza, designed to be paired with both fully detailed and time-averaged contact data. We show that the fully detailed, data-based transmission model produces influenza transmission results consistent with observed school outbreak data. Then we explore the effects of two types of simplifying assumptions about underlying contact networks on outbreak results.

First, we explore the implications of the dynamic nature of the contact networks. Pairs of students in close proximity change throughout the school day and from one day to the next. Contact networks underlying transmission models are commonly assumed to be static entities, and the precise timing of contacts is ignored [20]. However, contact timing details might better inform our understanding of transmission patterns. One study similar to ours found that aggregating contacts over 1 day did not affect simulated outbreak sizes [15], but, in different contexts, others found that static networks drastically failed to reproduce outbreak sizes and patterns exhibited by their dynamic counterparts [17,19].

Second, we explore the implications of heterogeneity of cumulative pairwise contact durations. Close-proximity contacts range from brief, single encounters to long, repeated interactions, presumably translating to heterogeneous pairwise transmission probabilities. Theoretical network transmission models often assume equal pairwise transmission probabilities, producing analytic results for relationships between topological network properties and expected outbreak properties such as initial rate of growth, final size and epidemic threshold effects [21]. We aim to better understand the applicability of this theory when the homogeneous transmission probability assumption is violated. Comparative simulation studies can shed light on this question; some have found that averaging contact durations caused significant increases in simulated outbreak sizes [15,17]. Another study based on survey data found much smaller effects from such averaging [22]. We add more comparative examples with this body of work and also provide insights into properties of weighted networks that could forecast the effect that an averaging assumption would have on final outbreak size.

## 2. Material and methods

### 2.1. Data collection from schools

We deployed wireless ranging enabled nodes (WRENs) [23] to students in Utah schools. Each WREN was worn by a student and used sensor technology to collect time-stamped data from other WRENs in proximity at intervals of approximately 20 s. Each recording included a measure of signal strength, which depends on the distance between and relative orientation of the pair of individuals wearing each WREN. Using test data from pairs of people standing face-to-face at distances of 1–4 m, we applied signal strength criteria for proximity such that each retained data point was most likely to represent a pair of students located 2 m or less from each other. Pairs of WRENs worn by students with non-face-to-face orientation or with a non-clear path between them would tend to have decreased signal strength [8] and would be less likely to be retained even within 2 m. We also performed a sensitivity analysis to test the effects of a stricter, 1 m criterion for contact data retention.

Here, we report on data we collected from two schools in Utah: one of the middle schools (Mid1), an urban public school with enrolment of 679 students, grades 7 and 8 (typical age range 12–14); and one of the elementary schools (Elem1), a suburban public school with 476 students, grades K–6 (typical age range 5–12). We captured valid, objective contact data during school hours of two consecutive school days in autumn 2012 from 591 students (87% coverage) at Mid1 and in winter 2013 from 339 students (71% coverage) at Elem1. We also linked gender, grade and class information to each WREN id. Coverage rates did not vary significantly by these sub-groupings (χ^2^-test of independence; *p* > 0.2 in each case).

The class schedule at Mid1 consisted of seven class periods, repeated each day, with students generally switching classrooms each period, and a lunch period. Students at Elem1 generally stayed in their respective classrooms during the school day, with the exception of lunch, recess and school assemblies or events. For further details of the data collection, and a description of the datasets provided, see the electronic supplementary material, datasets D1–D10.

### 2.2. Network analysis

From the raw data, we generated and analysed contact networks for each school. As detailed in the electronic supplementary material, we assumed that each WREN proximity record represented a face-to-face interaction of approximately 20 seconds, which was the interval between broadcasts from each WREN. Consolidating these interactions produced a dynamic network of student pairs who were in continual proximity at given time intervals. Aggregating the dynamic network produced a static network describing who contacted whom at least once during the observation period, with the total duration of contact for each pair across each day. We performed this processing using the software R [24], v. 3.0.1, and analysed the resulting networks using the R igraph package [25], v. 0.6.5–2, with specific equations and R commands detailed in the electronic supplementary material. Network measures are described and defined in the Results section.

### 2.3. Disease progression, transmission and location model

We used a susceptible–exposed–infectious–removed (SEIR) framework to dynamically classify simulated individuals. Under our assumption of a novel strain of influenza, all individuals were classified as susceptible to infection at the beginning of a simulation, with no immunity by vaccination. When simulated transmissions occurred, individuals moved to the exposed class, infected but not yet infectious. Individuals moved to the infectious class at the end of a random, exponentially distributed latent period with mean 0.54 days [26,27] (electronic supplementary material, table S1). During the infectious period, we applied time-varying levels of infectiousness following a log-normal distribution [28] (electronic supplementary material, table S2), truncated at 7 days and peaking 1.12 days after infectiousness onset [26]. Individuals in the removed compartment remained non-infectious and immune to re-infection.

The probability of transmission from an infectious to a susceptible individual depended on their duration of contact, the infectious person's infectiousness level during contact, and the overall transmissibility of the pathogen. The last was quantified by a single transmissibility parameter, defined as the product of the total amount of virus shed by the infectious person over the entire infectious period and the probability of infection per unit of virus shed by a contact in proximity, under an exponential dose–response model [29]. We derived the base case value of this transmissibility parameter from contact-transmission data [30]. We then assumed a higher level of transmissibility for network comparison. Under these assumptions, we derived an equation for the probability of transmission during each uninterrupted contact (see the electronic supplementary material).

We assumed that symptom onset of infected individuals occurred later than shedding onset. Specifically, infected individuals entered a symptomatic state after a random, log-normally distributed incubation period with a mean of 1.52 days and a standard deviation of 0.66 [31]. We correlated the latent and incubation period distributions, such that shedding began 0.5–1.2 days before symptom onset. However, under our assumptions for time-varying infectiousness, shedding amounts did not increase to an appreciable level until closer to the time of symptom onset.

We assumed uninfected students attended school during normal hours, Monday to Friday, every week throughout the simulation. Transmissions could occur only during school hours, while disease progression continued occurring through evenings and weekends. The symptom status model was used to trigger potential school absenteeism behaviour. Symptomatic individuals would enter a ‘stay-at-home’ state for a random, Poisson-distributed number of days, with mean 2 days [3]. Under this distribution, approximately 14% of infected students did not enter the stay-at-home state at all; the remaining students delayed leaving school up to 2 h after symptom onset, if onset occurred during school or just before school started. Upon exiting the stay-at-home state, students were returned to school at the beginning of the next school day, possibly while still infectious and symptomatic.

### 2.4. Networks for modelling contact durations

For each school, we tested up to eight different networks (table 1), which differed only in representation of the timing and duration of contacts. The *dynamic alternating network (D)* was the most detailed, retaining each time-stamped interaction recorded by the WRENs. Corresponding simulations alternated between the two daily dynamic networks on consecutive school days. The *static alternating network (S1)* retained only the total contact duration of each pair of students during each school day. Corresponding simulations alternated between the two daily static networks, assuming that contact pairs were in proximity with uniform probability, weighted by the total contact duration, throughout the school day. The *static averaged network (S2)* was constructed by retaining each pair of students who interacted at least once during the 2 days and applying the average of their two daily contact durations. This averaged network was then applied during each school day in the transmission simulations.

**Table 1.** Networks representing student contacts at schools.

network version	contact time aggregation (days)	contact duration change
dynamic alternating (D)	—	—
static alternating (S1)	1	—
static averaged (S2)	2	—
homogeneous (H)	2	average all
shuffled (Sh)	2	shuffle all
homogeneous by grade (HG)	2	average by grade
homogeneous by class (HC)	2	average by class
shuffled by class (ShC)	2	shuffle by class

The remaining five networks (table 1) retained the S2 assumption regarding contact timing and also changed pairwise contact durations. In the *homogeneous* (*H*) network, we applied the same duration to each pair, calculated to produce the same average probability of transmission across all edges as the averaged static network. In the *shuffled* (*Sh*) network, we randomly shuffled the pairwise durations across the network. For the three remaining networks, *homogeneous by grade* (*HG*), *homogeneous by class* (*HC*) and *shuffled by class* (*ShC*), we applied two separate sets of homogenized or shuffled durations across the network: one for edges that connected individuals in the same group (grade or class) and one for all other edges.

### 2.5. Outbreak simulations and output measures

Simulations were run using Anylogic 6 (XJ Technologies, St Petersburg, Russia), a Java-based modelling application. The program created an ‘agent’ representing each student in the school dataset and linked the agents together according to the contact data. We seeded each simulation with a single initial infectious individual, infected at a random time over a 7 day period. No additional infections were imported from outside the network after the initial one. Every individual in the school network was assigned the initial infection 1000 times each. The program assigned times with millisecond resolution for disease stage progressions, transmissions, leaving and entering school, etc. under our stated assumptions. Each simulation ran until no individuals remained infectious.

We averaged the expected number of transmissions from the initially infected individual over all simulations to arrive at *R*_0_, the within-school initial reproductive number, assuming each student was equally likely to import the initial infection. We also averaged the total outbreak sizes of each simulation. We calculated measures of variability in *R*_0_ and total outbreak size as two 95% ranges: first for the number of transmissions expected from each individual acting as the initial case (individual variability), and second for the actual total number of transmissions across every simulation (stochastic variability). Finally, we calculated the probability of an outbreak exceeding a given total after a single introduction, by each individual and averaged over all individuals.

## 3. Results

### 3.1. Data-based contact network

For Mid1, after cleaning the data and consolidating redundant proximity records, there were 614 104 proximity records across the two school days. We consolidated these records into 309 025 uninterrupted pairwise interactions and 56 867 unique pairs with at least one contact. At Elem1, we collected 292 653 proximity records, consolidated into 151 421 pairwise interactions and 16 546 unique contact pairs.

Aggregating these contact data over 1 day or both days produced weighted contact networks, with nodes representing students, edges connecting students with any contact and edge weights corresponding to the duration of contact per day. At Mid1, the 2 days produced remarkably similar network statistics (table 2); e.g. the network density (fraction of all possible pairs having at least one contact) was 0.22 on both days. Elem1 saw an increase in network density from day 1 (0.15) to day 2 (0.22), probably because of a school-wide science fair held during school hours on day 2. The combined, both-day network densities (0.33 for Mid1, 0.29 for Elem1) were higher than the 1 day networks, demonstrating that many contact pairings occurred on one day but not the other. While Elem1 had lower density than Mid1, it had longer average contact durations (2.9 versus 1.8 min per day). The squared coefficients of variation (CV^2^) for degree (number of different students contacted) and strength (total time per day spent in contact) were all less than 1, suggesting low variability in contact rates across different students, especially at Mid1.

**Table 2.** Student contact network measures. Elem1, elementary school; Mid1, middle school; node degree, number of unique other students contacted for any duration, across 1 day or both days, by a given student; node strength, total duration (minutes per day) of contact across all contacts; edge duration, total duration (minutes per day) of contact for a given contact pair; network density, fraction of all possible node pairs that had a contact of any duration; global clustering, the probability that a connected triple is part of a triangle; weighted clustering as defined in [32]. Mean shortest path length is the average minimum number of edges (of any duration) needed to connect a random pair of nodes in the network. Mean most probable path length incorporates edge durations to calculate the average number of edges along the most probable pathway of transmission between any two nodes, under a sample transmission scenario (see the electronic supplementary material, S1). Grade or class assortativity [33] quantifies the tendency for nodes in the same group to be connected to each other beyond (if positive) or below (if negative) what would be expected randomly. The weighted versions of assortativity use the total durations of within-group versus between-group edges rather than the number of edges.

	Mid1 day 1	Mid1 day 2	Mid1 day 1 and 2	Elem1 day 1	Elem1 day 2	Elem1 day 1 and 2
number of nodes	591	591	591	339	339	339
mean degree (CV^2^)	128 (0.07)	129 (0.08)	192 (0.04)	52 (0.12)	76 (0.17)	98 (0.12)
mean strength (CV^2^)	338 (0.14)	348 (0.16)	343 (0.11)	257 (0.26)	305 (0.66)	281 (0.33)
mean edge duration (min/day)	2.64	2.69	1.78	4.98	4.03	2.88
network density	0.22	0.22	0.33	0.15	0.22	0.29
global clustering coefficient	0.29	0.30	0.39	0.39	0.40	0.44
weighted clustering coefficient	0.34	0.35	0.43	0.55	0.54	0.56
mean shortest path length	1.78	1.78	1.67	1.99	1.81	1.72
mean most probable path length	2.44	2.43	2.23	2.60	2.29	2.11
grade assortativity	0.36	0.36	0.28	0.51	0.31	0.28
weighted grade assortativity	0.71	0.68	0.70	0.93	0.88	0.90
class assortativity				0.27	0.16	0.11
weighted class assortativity				0.83	0.81	0.82

Clustering coefficients measure the cohesiveness of local groups of nodes; the global clustering coefficient, or transitivity, is the probability that a connected triple (two edges connecting three nodes) also forms a triangle (i.e. the probability that two different contacts of a student also contacted each other). Each clustering coefficient was greater than the network density, so triangles occurred more frequently than expected in a random network of the same density. Clustering was higher at Elem1 (0.44 versus 0.39 for Mid1) despite having lower network density. Those values do not incorporate edge weights; the weighted clustering coefficient [32] determines the extent to which higher edge weights of a connected triple increase the likelihood of being part of a triangle. Weighted clustering was higher than global clustering, which means that triangles were more likely to occur among contact pairs with higher contact durations. Again, this effect was stronger at Elem1 (0.56 versus 0.43 at Mid1).

The mean shortest path lengths were low for all networks; less than two steps on average were required to travel from one node to any other node. As many of these shortest paths could traverse edges with very low durations and thus low transmission probability, we also calculated the average ‘most probable path length’ (electronic supplementary material), which is the length of the most likely chain of transmission from one node to another. These values were also low (less than 3 for all networks), suggesting that the weighted networks can also be characterized with this small-world property.

Assortativity [33] by group compares the number of between-group and within-group contacts with the expectation if groups did not influence contact behaviour. We found positive assortativity by grade (and class for Elem1) in each network, which means that students had more contacts with students in the same group than expected randomly. Elem1 showed higher grade and class assortativity on day 1 (0.51 versus 0.31 on day 2), probably because of a multi-grade school science fair occurring on day 2. Under weighted assortativity, which measures total duration of within- and between-group contacts, this difference was much less pronounced (0.93 versus 0.88), implying that between-group contacts at the science fair were of short duration. Across both days, grade assortativity was the same at each school (0.28) but stronger at Elem1 when weighted (0.90 versus 0.70).

We recalculated network statistics (electronic supplementary material, table S3) after applying a stricter criterion for retained WREN signal strength, eliminating contacts likely to be greater than 1 m distant rather than 2 m. This eliminated approximately 43% and 47% of the edges in the Mid1 and Elem1 both-day networks, respectively. However, key features and comparisons described above were robust to this change, including low variability in degree and strength, low mean shortest path lengths, and higher clustering and grade assortativity, both weighted and un-weighted, at Elem1 compared with Mid1.

### 3.2. Within-school *R*_0_

Under the most detailed network data (version D) and disease progression and transmission assumptions based entirely on influenza data, we calculated *R*_0_, the expected number of within-school transmissions from an initially infected individual. For Mid1, we found *R*_0_ = 0.79 (95% individual variability 0.30–1.35; stochastic variability 0–4). For Elem1, we found *R*_0_ = 0.62 (0.15–1.24; 0–4). We calculated adjustments to these values to account for non-participating students at each school, by assuming that the contact network including all enrolled students would have the same density as the network of valid study participants, and that the average transmission probability per contact would be the same for the missing contacts with and among non-participants. These adjustments led to *R*_0_ = 0.91 for Mid1 and *R*_0_ = 0.87 for Elem1 (table 3).

**Table 3.** Within-school *R*_0_ results under different contact distance criteria. *R*_0_ values from transmission simulations across four different contact networks, using influenza transmissibility and disease progression time course assumptions derived entirely from data. *R*_0_ values were derived from averaging the expected number of transmissions from each initially infected individual across all simulations, with each student being assigned the initial infection 1000 times. Adjusted values were calculated by assuming that the contact network including all enrolled students would have the same density as the network of valid study participants, and that the average transmission probability per contact would be the same for the missing contacts including non-participants. The *R*_0_ values for each school under the 2 m distance criterion are more consistent with an independent estimate of 0.9 (95% CI 0.7, 1.1) for the within-school component of *R*_0_ during an influenza outbreak at an elementary school [34].

contact distance criterion	Mid1 *R*_0_ (adjusted)	Elem1 *R*_0_ (adjusted)
2 m	0.79 (0.91)	0.62 (0.87)
1 m	0.23 (0.26)	0.22 (0.31)

We also recalculated these values under the network resulting from the stricter transmission distance assumption of less than 1 m instead of less than 2 m, resulting in *R*_0_ = 0.23 (0.08–0.42; 0–2) for Mid1 and *R*_0_ = 0.22 (0.04–0.46; 0–2) for Elem1 (table 3). Non-participant adjustments resulted in *R*_0_ = 0.26 for Mid1 and *R*_0_ = 0.31 for Elem1. The 2 m distance criterion results are more consistent with an independent estimate of 0.9 (95% CI 0.7, 1.1) for the within-school component of *R*_0_ during an influenza outbreak at an elementary school [34]. Therefore, we chose to retain the network resulting from the 2 m distance criterion for the remaining analyses.

### 3.3. Outbreak simulations for network comparisons

**Figure 1.**
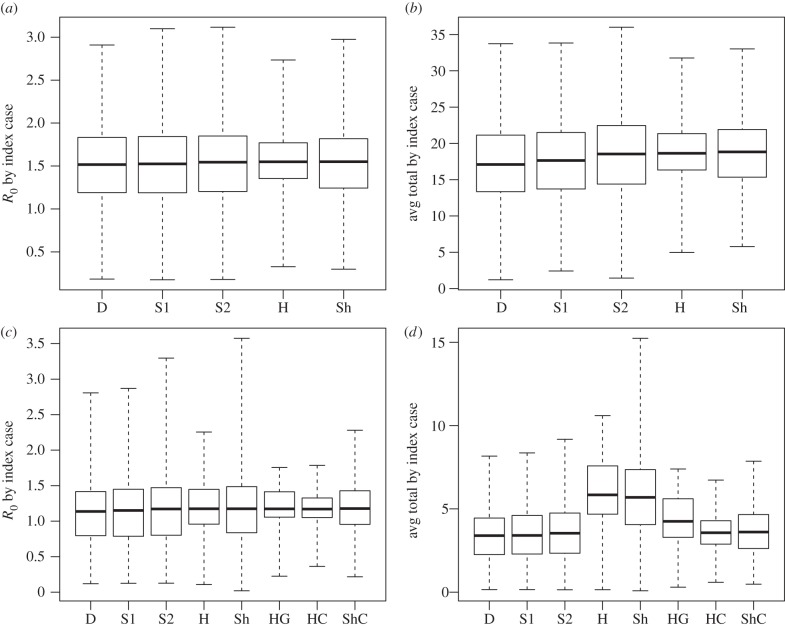
Variability in transmission results across initially infected individuals. Comparison of variability across initially infected individuals in the number of transmissions using the Mid1 network (*a*,*b*) and the Elem1 network (*c*,*d*) under selected versions of our simulations, with higher-transmissibility parameter (*k* = 24). Each box plot represents statistics over each individual in the network (591 individuals for Mid1, 339 for the Elem1), averaged over the 1000 simulations for each individual. Solid line, average (as listed in table 4); box hinges, interquartile range; whiskers, full range. (*a*,*c*) Average transmissions from the initially infected individual only (solid lines represent population-wide *R*_0_). (*b*,*d*) Average total number of transmissions when each individual imported the initial infection. D, dynamic alternating; S1, static alternating; S2, static averaged; H, homogeneous transmission probabilities; Sh, shuffled transmission probabilities; HG, homogeneous by grade; HC, homogeneous by class; ShC, shuffled by class.

**Figure 2.**
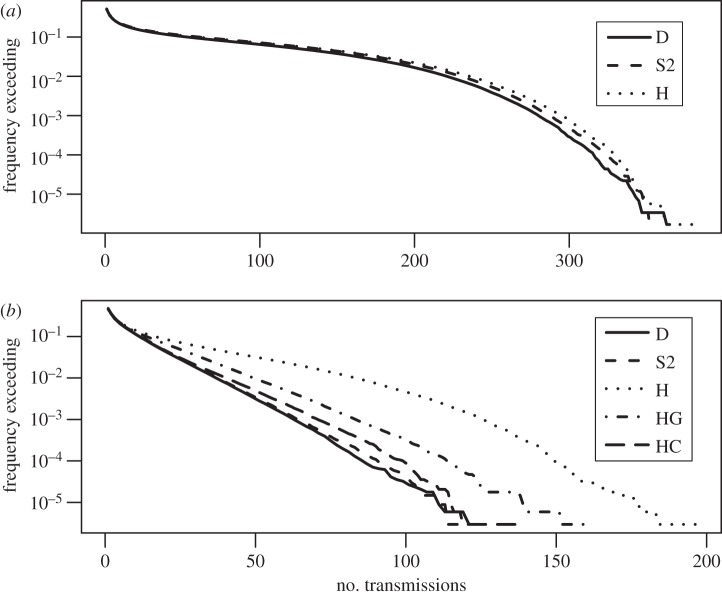
Overall variability in transmission results. Reverse cumulative distribution functions comparing the overall variability in total number of transmissions using the (*a*) Mid1 and (*b*) Elem1 networks, with higher transmissibility (*k* = 24), under selected versions of our simulations. Curves show the proportion of simulations (591 000 for Mid1; 339 000 for Elem1) in which the total number of transmissions exceeded the given number. D, dynamic alternating network; S2, static averaged network; H, homogeneous transmission probabilities; HG, homogeneous by grade; HC, homogeneous by class. The following curves were removed for visual clarity: version S1 (static alternating) produced very similar curves to D and S2 at both schools; version Sh (shuffled transmission probabilities) was similar to H at both schools; and version ShC (shuffled by class) was similar to HC in (*b*).

With *R*_0_ < 1, outbreaks from a single introduction are rare and tend to be small when they do occur, especially within a small, clustered population in which susceptible contacts can be depleted quickly. Given that the dynamics of larger outbreaks can better illuminate the effects of network structure on transmission, and given that the transmissibility characteristics of a novel strain of influenza are subject to considerable uncertainty, we chose to double the transmissibility parameter from its data-based value for comparing simulations across different networks, to achieve *R*_0_ in the range 1–1.5 at each school. Under version D for Mid1, this resulted in *R*_0_ = 1.5 (unadjusted) and an average total outbreak size of 17.1 infected students. For Elem1, the result was *R*_0_ = 1.1 and an average total of 3.4. For comparison, we calculated the results from all networks for *R*_0_, average total outbreak size, and variability in these values across the identity of the initial infection and across all simulations (table 4, figures 1 and 2).

**Table 4.** Comparison of transmission simulations results by assumed network. Mid1, middle school; Elem1, elementary school; *R*_0_ is the expected number of transmissions from the initially infected individual only, with variability expressed as two different 95% ranges: first as the range of the expected number of transmissions from the initial individual depending on which particular individual in the network was the initial case, and second as the number of actual transmissions from the initially infected individual across every simulation; average total is the average total number of transmissions until the end of each simulated outbreak, with variability expressed as 95% ranges, first for the average number of total transmissions depending on which individual in the network is the initial case, and second for the actual total number of transmissions across every simulation; increases are expressed as the percentage increase in the average total compared with version D for the same school. *R*_0_ for versions S2 and all homogeneous and shuffled versions are the same according to our assumptions (electronic supplementary material); slight differences in those results are due to stochasticity in model runs.

	*R*_0_ (variability)	average total (variability)	avg. total increase (%)
Mid1 networks			
dynamic alternating (D)	1.52 (0.6 to 2.5; 0 to 7)	17.1 (5.7 to 28.0; 0 to 178)	—
static alternating (S1)	1.52 (0.6 to 2.6; 0 to 7)	17.6 (6.3 to 28.8; 0 to 182)	+3
static averaged (S2)	1.54 (0.6 to 2.5; 0 to 7)	18.5 (6.3 to 31.1; 0 to 188)	+8
homogeneous (H)	1.55 (0.9 to 2.2; 0 to 7)	18.6 (10.7 to 25.5; 0 to 193)	+9
shuffled (Sh)	1.55 (0.7 to 2.5; 0 to 7)	18.8 (9.5 to 28.5; 0 to 191)	+10
Elem1 networks			
dynamic alternating (D)	1.14 (0.3 to 2.2; 0 to 6)	3.39 (0.4 to 6.6; 0 to 26)	—
static alternating (S1)	1.15 (0.3 to 2.2; 0 to 6)	3.41 (0.4 to 6.7; 0 to 26)	+0.4
static averaged (S2)	1.17 (0.3 to 2.2; 0 to 6)	3.54 (0.4 to 7.2; 0 to 27)	+4
homogeneous (H)	1.18 (0.3 to 2.0; 0 to 6)	5.85 (1.0 to 9.5; 0 to 57)	+72
shuffled (Sh)	1.17 (0.2 to 2.1; 0 to 6)	5.69 (0.7 to 10.2; 0 to 54)	+68
homogeneous by grade (HG)	1.17 (0.3 to 1.7; 0 to 6)	4.26 (0.5 to 6.8; 0 to 35)	+25
homogeneous by class (HC)	1.17 (0.5 to 1.6; 0 to 6)	3.57 (0.7 to 5.9; 0 to 27)	+5
shuffled by class (ShC)	1.18 (0.4 to 1.9; 0 to 6)	3.61 (0.6 to 6.7; 0 to 27)	+6

#### 3.3.1. Dynamic versus static

**Figure 3.**
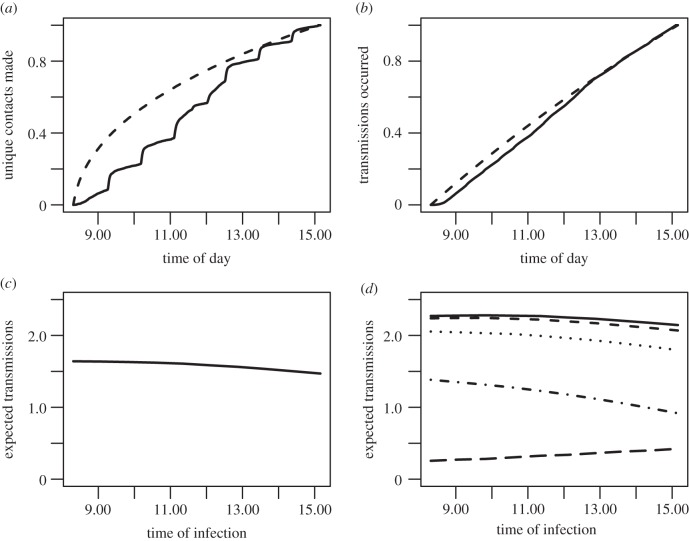
Comparison of transmission results on the dynamic and static networks. (*a*) The proportion of unique contacts made by time of day in the dynamic contact data (solid) compared with those expected under the static network assumption (dashed). The solid curve clearly exhibits the effects of the class-switching schedule of the school, while the dashed curve shows that more unique contacts would be made earlier in the day if the same number of interactions were distributed randomly. (*b*) From the transmission simulations, the cumulative proportion of all transmissions that occurred by the given time of the school day in the dynamic alternating (solid) and static alternating (dashed) versions. The static assumptions cause transmission times to shift earlier in the day. (*c*) The expected number of transmissions from an individual who was infected at a given time of the school day, averaged over the five school days Monday–Friday. Results are from the Mid1, static averaged network, higher-transmissibility scenario. (*d*) Expected transmissions by time of day for each day Monday–Friday (top to bottom): solid, Monday; short dash, Tuesday; dot, Wednesday; dash-dot, Thursday; long dash, Friday. Individuals infected Monday–Thursday morning will be more infectious themselves during school the following day than those infected in the afternoon, because the peak of infectiousness is not typically reached until more than a day after exposure. By contrast, individuals infected on Friday will usually be past peak infectiousness when returning to school 3 days later on Monday, so those infected more recently (i.e. Friday afternoon) will produce more transmissions on average. The overall downward daily trend in expected transmission occurs because the weekend occurs during a greater area of the infectiousness curve the later in the week one is infected.

For both schools, averaging contact timing over each day, version S1, or both days, version S2, produced 1–8% increases in average total transmissions compared with version D (table 4), and they produced similar variability (figures 1 and 2). Version S1 assumptions caused contacts to shift earlier in the school day, on average, than when they actually occurred; i.e. new contacts occurred earlier than expectation if the same total number of contacts were made at random, uniformly distributed times (figure 3*a*). This contributed to causing transmissions to occur earlier in the school day in S1 simulations (figure 3*b*). We then used the Mid1, S1 simulation results to plot the expected number of transmissions from initially infected individuals against their own time of infection. Being infected earlier in the school day tended to increase one's probability of infecting others on subsequent days (figure 3*c*), an effect which holds for every individual day except Friday (figure 3*d*), because an individual infected on Friday was expected to be past peak infectiousness by the time school returned on Monday. The effect of the day of infection (Monday–Friday) on within-school transmission potential was due to the weekend overlapping with the peak infectiousness of students acquiring infection later in the week, and not due to differences in observed contact patterns on different days of the week. This kind of weekend effect has been shown to play a role in shaping incidence curves during observed influenza outbreaks [35].

The 2 day averaging window of version S2 began to overlap with the distribution of serial intervals (electronic supplementary material, figure S1), the time between infection of and subsequent transmission from an individual. While serial intervals occurring during one school day were extremely rare, next-day intervals were fairly common, accounting for 16% of transmissions on the D, Mid1 network (an additional 14% occurred Friday–Monday). Many student pairs had contact on one of the two deployment days but not the other, information that was lost when averaging over both days. Thus, version S2 allowed chains of transmission across consecutive school days that were not possible under version S1, which probably contributed to the increase in outbreak sizes. However, the effect of this phenomenon on overall transmission was just an additional 3–5% increase in average outbreak size.

#### 3.3.2. Homogeneous/shuffled contact durations

Next we explored the effects of assuming homogeneous contact durations (version H) and shuffling durations across all contact pairs (version Sh). These changes did not affect *R*_0_ because they do not alter the total transmission probability across all edges in the network, and *R*_0_ depends only on this total as we have defined it (electronic supplementary material). The effects on total transmissions, however, were different depending on the school (table 4). For Mid1, versions H or Sh caused a 1% increase in average transmissions compared with version S2, and less than 10% increase compared with the original version D. For Elem1, there was a much more substantial increase in transmissions under versions H and Sh (72% and 68%, respectively, compared with version D). Using separate homogeneous durations for contacts within and between grades (HG) also produced a relatively substantial 25% increase, but doing the same at the class level (HC) as well as shuffling durations at the class level (ShC) brought the results in line with version S2.

The simulations showed variability in *R*_0_ and total outbreak size across different identities of the initial infection, which was reasonably well maintained across the different networks for Mid1, but less well for Elem1 (figure 1). For overall variability in outbreak sizes across all simulations, all versions for Mid1 produced similar distributions to the baseline (figure 2*a*), while for Elem1, changing the distribution of contact durations caused a higher probability of large outbreaks (figure 2*b*).

The homogeneous durations applied in our H, HG and HC simulations (table 5) help explain why version H produced larger school-wide outbreaks at Elem1. Applying the all-network homogeneous duration to edges connecting students in different grades or classes tends to drastically increase the transmission probability across those edges, which causes large school-wide outbreaks to become more probable. For example, the average transmission probability across all contact pairs was about 1%, but only 0.2% across contact pairs from different grades and 6% across contact pairs in the same class. While the HG assumption produced suitably low probabilities of transmission between students in different grades, it still increased the transmission probability between students in the same grade but different classes.

**Table 5.** Average transmission probabilities within networks. We calculated transmission probabilities for each edge in the network during the S2 simulations, and then averaged them accordingly. The homogeneous durations were then calculated to determine which duration, if applied to every edge, would produce the correct average probability. These homogeneous durations are not equal to the average contact durations, because the relationship between contact duration and transmission probability is nonlinear. For Elem1, the within-group probabilities and associated durations are substantially larger than those between groups and overall.

school	edge category	average transmission probability per edge	homogeneous duration (min per day)
Mid1	all	0.00759	1.69
Elem1	all	0.0114	2.54
Elem1	within grade	0.0262	5.94
Elem1	between grade	0.00170	0.37
Elem1	within class	0.0555	13.02
Elem1	between class	0.00262	0.58

## 4. Discussion

### 4.1. Novel contact data and influenza transmission model

Our new contact datasets add to a growing body of objective proximity data from schools [8–13] and are the first from a middle school and an American elementary school (excepting lunchtime-only interaction data among sixth graders [12]). Our influenza transmission model is a novel contribution that is complex enough to explore the implications of detailed features of contact data and simple enough to be parametrized transparently with influenza data wherever possible. Our most detailed, dynamic networks combined with data-based influenza parameter values produce our best predictions of transmission (table 3) for a novel strain of influenza spreading among students within school hours. Our adjusted *R*_0_ values of 0.91 and 0.87 are remarkably similar to the estimate of 0.9 for the within-school component of *R*_0_ from a detailed investigation of an outbreak of 2009 influenza A/H1N1 at an elementary school [34]. That study's estimate of *R*_0_ from students was 1.7 when including transmissions that probably occurred outside of school, which is similar to other estimates for 2009 H1N1 [36,37]. While we have not included such outside-school contacts in our transmission model, the within-school component of overall transmission is important, especially when considering the effects of school closures as a public health intervention. Our results show that our assumptions about relevant within-school contacts and their relationship to influenza disease progression and transmission lead to realistic results for this component.

Our model is amenable to improvements as further quantitative knowledge is gained about influenza infection and transmission mechanisms and absenteeism behaviour of symptomatic students. It also can be easily calibrated by adjusting a single parameter for transmissibility. We did so by doubling this value to produce new sets of simulated outbreaks with *R*_0_ > 1 (1.5 and 1.1 for Mid1 and Elem1), which more often consist of larger outbreaks with several generations of transmission and are still reasonable assumptions for a novel strain of influenza with uncertain transmissibility characteristics. We used this higher-transmissibility scenario to emphasize the effect of contact heterogeneity on outbreak size. In preliminary analyses, the general effects of heterogeneity we describe were also present but less obvious under the lower data-based transmissibility.

### 4.2. Effect of heterogeneity in contact timing

Averaging fluctuations in contacts during a school day by using a static network caused a 1–3% increase in average outbreak sizes. A common explanation for such an increase is that static networks allow multiple-generation transmission chains along paths that are unlikely or impossible in corresponding dynamic networks. However, multiple generations of transmission during a single school day were extremely rare in our simulations. The assumption of uniform contact timing tended to shift transmissions earlier in the school day and, under our influenza model, those infected earlier in the day were more likely to transmit to others if school was held the following day. This potential effect of time-averaging has not been identified in previous modelling studies, to our knowledge. However, if a 1–3% change in average outbreak size is acceptable, then intra-day contact network fluctuations can be ignored for simplicity, a conclusion that is consistent with another result based on contact data from academic conference attendees [15].

Averaging contact timing over 2 days caused an additional increase in outbreak size compared with averaging over each day separately. Here, because 2-day serial intervals occurred relatively frequently under our model, the 2-day averaging allowed more possible transmission chains on consecutive school days. However, the 2-day averaged static model still produced results within 10% of average outbreak sizes compared with the fully dynamic model.

In light of the results of both versions of the static network, we conclude that the temporal patterns of contacts over 2 days or less may not be of critical relevance for predicting the potential size of influenza outbreaks over these networks. By contrast, a school-epidemic model with instantaneous transmission on contact, no recovery, and no latent or incubation period (suitable for modelling, say, the spread of rumours in a school) concluded that temporal structure of contacts has a more significant influence on simulation outcomes than what we have found here [38]. Epidemic models over networks in other settings have also found that aggregating temporal networks into static networks can increase or decrease outbreak size and timing [17,19,39]. Whether aggregating a school contact network across several days or weeks (allowing multiple generations of transmission to occur during the aggregated period) would produce results similar to these studies is an area for future work.

We do not imply that intra-day contact patterns are unimportant for the issue of school transmission of influenza and similar diseases. Studying the contributions of individual periods of a school schedule to the structure of the overall school contact network may be critical to understanding why some schools might be more vulnerable to large outbreaks and what schools might be able to change about their schedules, short of closure, to reduce risk. Our dynamic network data combined with the data we collected on school schedules can support this promising area of future work.

### 4.3. Effect of heterogeneity in contact duration

Our data also showed wide heterogeneity in cumulative pairwise contact durations. Previous transmission network modelling studies have equated or averaged transmission probabilities across all assumed contact pairs, focusing on studying the topology of the resulting un-weighted networks. We have investigated whether the heterogeneity observed in our data can interact with network topology to affect the expected total size of an outbreak.

For the Mid1 network, averaging transmission probabilities resulted in only slightly larger outbreak sizes, suggesting that knowing only who contacted whom would mostly suffice to predict outbreak sizes at this school, as the added information of the distribution of contact durations did not substantially change those results. If this result generalizes to similar schools, it could lead to useful simplifications for both theoretical modelling work and practical risk assessment. Network transmission theory is better developed for un-weighted compared with weighted networks, and this theory might be useful for school outbreak preparation.

By contrast, averaging transmission probabilities resulted in substantial increases in outbreak size on the Elem1 network. Elem1 students stayed with their class for most of each school day. While inter-class contacts did occur, they had significantly shorter average duration than contacts between classmates. Inter-class contacts were important for school-wide transmission because they were the only route by which infection could spread from one class to another. When contact durations were homogenized, these critical inter-class contacts were given undue weight, causing larger outbreaks to become more likely. Randomly shuffling the observed contact durations around the network caused a similar increase, further illustrating the importance of the way contact durations are distributed in relation to other important network features. To test whether the science fair that occurred on the second day of data collection at Elem1 affected this conclusion, we re-ran the simulations using only data from day 1, and the homogeneous-duration assumption still caused a substantial increase in outbreak size.

Classifying each Elem1 contact into intra- or inter-class categories and applying a separate homogeneous transmission probability calculation across each category produced a network that adequately reflected the transmission results of the original. From this result, we conclude that classroom structure was the most important factor in determining the effects of contact duration heterogeneity on transmission in this school. While there was also considerable variation in contact durations within each of the intra- and inter-class groups, this within-group heterogeneity did not substantially influence outbreak size.

The results for Mid1 and Elem1 differed substantially in the effects of averaging edge weights on transmission results. Similar tests on contact networks in other settings have also found differing effects [15,17,22]. Although we have only two schools to compare here, some network statistics (table 2) show promise as predictors of the effects of such averaging. Comparing statistics on edge-weight-independent topological network features with their weight-dependent counterparts gives a sense for the effects of averaging edge weights, as doing so eliminates the effects of edge heterogeneity. For example, it has been established that increased network clustering, all else equal, tends to decrease the likelihood of large outbreaks over a contact network [40]. When comparing the clustering coefficients of our school networks with their weighted counterparts, we see that the difference at Elem1 was greater (0.56 weighted versus 0.44 un-weighted) than the difference at Mid1 (0.43 versus 0.39).

Less abstractly, assortativity by groups also has potentially important implications for school transmission, with higher assortativity decreasing the likelihood that an outbreak will spread outside of the group in which it started. Weight-independent grade assortativity was very close for both schools (0.28), but weight-dependent assortativity was higher at Elem1 (0.90 versus 0.70 at Mid1). Furthermore, Elem1 contained both more grades and smaller average grade size, which means that high-grade assortativity would have a stronger effect in limiting absolute outbreak size at Elem1. Finally, Elem1 can be broken down into even smaller groups of classes, and weighted assortativity by class was also very high: 0.82, compared with only 0.11 for the weight-independent version of the statistic. These statistics hold intuitive meaning in the light of potential strategies for low-disruption interventions, such as targeted class or grade closures [41], in the early stages of a novel influenza outbreak. Our results suggest that such interventions would be less effective at schools with contact structure similar to Mid1.

### 4.4. Limitations

We included only a portion of school-age children's daily and weekly contacts. Given that many contacts occurred on one day but not the other, additional days of data probably would have added still more contact pairs to the aggregated network. The implications of this possibility could be investigated by generating different realistic daily networks on each school day in transmission simulations. Including contacts outside of school hours also might affect outbreak patterns, especially if substantial contact occurs between students who did not interact at school. For example, transmissions between siblings attending the same school might be an important pathway for between-grade spread that we did not capture, perhaps attenuating the grade- and class-based assortativity that drove our Elem1 results. Alternatively, including non-school contacts might accentuate those results, if classmates engage in substantial mixing outside school.

The WRENs recorded contact only between study participants and were subject to imperfect capture rate and imperfect specificity to the desired pairwise distance and orientation ranges. The contact data do not address influenza transmissions that may occur over longer distances through airborne or fomite transmission, nor do they capture whether a contact involved direct touching or talking; combining WREN data with survey and location data may address some of those issues [42]. Data may have been affected by students removing or handling the WRENs or otherwise behaving atypically due to the study.

Our transmission model assumptions have limitations where data are lacking, especially (a) the relationship between contact duration and transmission probability, (b) individual heterogeneity in susceptibility, shedding, disease progression and symptomaticity, and (c) absenteeism or other contact-altering behaviour among symptomatic students. Of these, assumption (a) has a particularly strong effect on our conclusions. We used data from an isolated incident of transmissions occurring on an airliner [30] to arrive at the key parameter value, which is of questionable relevance to other settings and requires large extrapolations to predict transmission probabilities for short-duration contacts. If our extrapolation assumptions are violated, perhaps leading to a nonlinear, more threshold-like relationship between contact duration and transmission probability, then short-duration contacts are less relevant. For example, shorter-duration interactions could be less likely, per unit time, to involve higher-risk interactions such as touching or face-to-face conversation. However, the fact that our assumptions led to influenza transmission results consistent with observed outbreaks is reassuring.

We have not formally tested sensitivity of our results and conclusions to influenza-specific assumptions such as the time course of disease progression. However, we expect our main conclusion about the differing effects of heterogeneous contact duration between Mid1 and Elem1 would still hold for diseases with different time courses, because this was primarily an effect of the underlying static contact networks. The results regarding contact timing would probably be sensitive to disease time course assumptions, as results for faster-progressing diseases would be more affected by time-averaging contacts.

## Supplementary Material

Supplementary Material

## Supplementary Material

Dataset D1

## Supplementary Material

Dataset D2

## Supplementary Material

Dataset D3

## Supplementary Material

Dataset D4

## Supplementary Material

Dataset D5

## Supplementary Material

Dataset D6

## Supplementary Material

Dataset D7

## Supplementary Material

Dataset D8

## Supplementary Material

Dataset D9

## Supplementary Material

Dataset D10

## Data accessibility

The datasets supporting this article have been uploaded as part of the electronic supplementary material.
