# Supplementary material for: The role of heterogeneity in contact timing and duration in network models of influenza spread in schools
Source: J R Soc Interface. 2015 Jul 6;12(108):20150279. doi: 10.1098/rsif.2015.0279 (PMC4528592; doi:10.1098/rsif.2015.0279)
Supplement: Supplementary Material [file rsif20150279supp1.pdf]

Electronic Supplementary Material

S.1 Supplementary Methods

S.1.1 Schools

We deployed Wireless Ranging Enabled Nodes (WRENs) [23] to students in two Utah schools, an urban middle school (Mid1) on 11/28 and 11/29/2012 and a suburban elementary school (Elem1) on 1/31 and 2/1/2013. At Mid1, school hours were 8:25am to 3:15pm on each day. At Elem1, school hours were 8:30am to 2:45pm on each day for all students except kindergarteners, for whom school hours were 8:30 to 11:05am or 12:10 to 2:45pm. Each school was located in a single building with adjacent outdoor areas. Mid1 students were 7th and 8th grade (typical age range 12 to 14), and the school schedule consisted of seven class periods, with students generally switching classrooms between periods, in addition to two non-overlapping lunch periods. Aside from lunch, there were no other scheduled large-group activities or gatherings at Mid1. At Elem1, students generally stayed with their assigned class throughout the day; there were 21 different classes across the seven grades (K-6, typical age range 5 to 12). Seating arrangements in the classrooms at Elem1 may not have been the same across the two deployment days, as teachers at this school generally change their classroom seating arrangements at the end of a calendar month. Different classes at Elem1 had the potential opportunity to mix during lunch, recess, an assembly during the morning of the first deployment day and a science fair during the afternoon of the second deployment day. To our knowledge, the weather on the deployment days of each school did not prevent students from going outside during the school day.

S.1.2 Data Collection

At Mid1, WRENs were distributed to students in their first-period classrooms and collected before the end of last period on each day. At Elem1, WRENs were distributed near the start of school on day 1 and collected at the end of school on day 2 (students kept nodes overnight between the two school days, although only data from school hours were used in the analyses for this paper). At distribution, the students, assisted by teachers and our deployment team, filled out a log sheet with the ID on their node so that data could later be linked to individual students. In each classroom, teachers recorded the time that distribution and collection ended in their class and also recorded the IDs and in- or out-times for individual nodes that were distributed later or collected earlier than the rest of the class. Students were asked to attach a WREN to their clothing and wear it throughout the day.

Each node was programmed to broadcast a data packet at intervals of approximately 20 seconds. Precisely, the broadcast interval was programmed to be 20,000 binary milliseconds, which equals 19.53125 seconds (there are 210 = 1024 binary milliseconds per second). Each packet of information contains the ID of the node sending the signal and the time the broadcast was sent. The global times for all nodes were synced the day before each deployment began during a time sync process. Data from the time sync process were used to convert the nodes' time data to the true date and time of day that each data packet was broadcast and received.

All nodes were also programmed to record data packets broadcasted by other nodes. Nodes were always available to receive data, so a node could receive and record packets from multiple other nodes in quick succession. Upon receiving a packet, a node would record the information from the sender as well as its own measure of the global time, in addition to a radio signal strength indicator (RSSI) that is a measure of the strength of the signal received from the sender. While the nodes had been pre-programmed to ignore packets with low signal strength (to minimize recording of long-distance contacts), further testing after the deployments revealed that packets were frequently being retained from senders at a distance of greater than 2 meters from the receiver. Using data from these tests, we chose to ignore packets of data associated with an RSSI less than 14, which corresponds to a signal strength of −77 dBm. After applying this cutoff, there still may have been contacts of greater than 2 meters that were retained, as well as contacts less than 2 meters that may have been discarded.

We applied a series of cleaning procedures to eliminate or fix faulty data, further detailed elsewhere [23]. First, we compared the measures of global time from the sender and receiver at each record to ensure the time synchronization had been retained. Through this process, we identified a minority of nodes that had experienced significant time drift due to imprecise timekeeping, and used their pairings with properly synchronized nodes to adjust incorrect times. Second, we searched for unusual spikes in contact rates and high signal strengths near the beginning and end of deployments, which indicated that some nodes had been distributed later and/or collected earlier than what was manually recorded by the teachers in classrooms. These anomalies were deleted to avoid including data from nodes that were not being worn (nodes sitting together in bags or boxes would record large amounts of high-signal-strength data that could significantly skew our results if retained).

After these adjustments, we used the following procedure to use the raw data to estimate the start times and durations of uninterrupted pairwise interactions. First we broke time-of-day into time bins of length 19.53125 seconds (the interval between node broadcasts), and each time record was placed into the appropriate bin. If a pair of nodes each had records in the same bin (i.e., if node A recorded a packet sent by node B and B recorded a packet sent by A within a few seconds of each other), then they were considered redundant and consolidated. If a given pair of nodes had n consecutive bins filled, then we assumed that the two students had an interaction (i.e., were in face-to-face proximity with each other) for n*19.53125 seconds, starting at the time of the first bin of the sequence. This consolidated form of the data was used to generate network statistics and served as the input to transmission simulations. This consolidation did not prevent us from recording a student having contact with multiple other students during the same time bin.

The contact data on which the main results of our manuscript were generated consist of four tabular text files (electronic supplementary material datasets D1-D4), one for each of two days at each of the two schools. These files consist of four columns and one row for each uninterrupted pairwise contact during that school day. The first two columns (labeled id1 and id2) identify the two students; the third column (labeled startTime) is the time of day the contact began, in units of 19.53125 seconds (since midnight); the fourth column (labeled duration) is the duration of the uninterrupted contact, in units of 19.53125 seconds.

Information about the students represented in the contact data is also provided (electronic supplementary material datasets D5-D6). The column labeled "id" corresponds to the id's found in the contact data. The column labeled "grade" is the school grade of the student (0 = kindergarten, 1 through 12 = 1st grade through 12th grade, -1 = unknown). The column labeled "class" (Elem1 only) is the class group of the student. The column labeled "gender" provides the gender of the student if known (0 = male, 1 = female, -1 = unknown).

Finally, the contact data used in the sensitivity analysis are also provided (electronic supplementary material datasets D7-D10). These are alternate versions of the contact data, as described above, in which a stricter criteria corresponding to <1 meter proximity was applied, as opposed to the <2 meter criterion used for the main results.

S.1.3. Network Measures

The following definitions and formulas were used to calculate the network measures shown in Table 2. We also specify commands used in R [24] version 3.0.1 and the R igraph package [25] version 0.6.5-2. Degree and strength were defined in the main text and calculated using the R/igraph commands "degree" and "graph.strength." Density was calculated as $N_e = (N_v(N_v - 1)/2)$, where N_e is the number of edges and N_v the number of vertices (nodes) in the network. CV^2 is the coefficient of variation squared, where CV is the standard deviation divided by the mean. The global clustering coefficient is defined as 3 times the number of triangles divided by the number of unique connected triples, calculated by the R igraph command "transitivity" (type "global"). The weighted clustering coefficient is defined by Barrat [32] , calculated using the R/igraph command "transitivity" (type "weighted"). Mean shortest path was calculated using the R/igraph command "average.path.length." The mean most probable path length calculation required that we first convert edge durations into transmission probabilities. To do this, we assumed that an uninterrupted infectious–susceptible interaction of the given duration occurred starting one day after the onset of shedding of the infectious individual, and used the base case transmissibility parameter $k = 12$ (see influenza transmission model equations below). Then we transformed these probabilities p using the expression $w = -\ln(p)$ and, using edge weights w as costs, calculated the average length of lowest-cost paths between each pair of nodes as determined by the R/igraph command "get.shortest.paths," which used Dijkstra's algorithm. Assortativity by grade/class/gender was calculated using the R command "assortativity.nominal," which uses a formula from Newman [33] (equation 2 in that paper). To calculate weighted assortativity, we used the same equation but changed the quantities e_{ij} to be the fraction of total network edge durations found on edges connecting nodes of type i to nodes of type j.

S.1.4. Influenza Progression and Transmission Model

We developed a new mathematical model for influenza disease progression with time-varying infectiousness. First, we quantified a distribution for the latent period, the time between infection (transmission) and onset of shedding. An experimental human data set for the onset of shedding after exposure [26, 27] is reproduced in Table S1. Because these data are well-fit parsimoniously by the exponential distribution, we chose this distribution rather than a less parsimonious two-parameter

distribution such as log-normal, and because the exponential distribution is a common assumption for the length of the "Exposed" period in commonly used SEIR differential equation models. In addition, another study [35] applied a technique to estimate the latent period from influenza outbreak data and found results consistent with an exponential fit to the data in Table S1. Using maximum likelihood estimation, we calculated rate parameter *r* = 1.8358 per day. Then, the probability that shedding begins before time *t* (days) is $1 - \mathrm{e}^{-rt}$.

For the incubation period, the time between infection (transmission) and onset of symptoms, we use the lognormal distribution defined in [31], with parameters μ = ln(1.4) and σ = ln(1.51). We assume that the latent period is not independent of the incubation period. When drawing random values, we use the same random seed for both the latent and incubation period such that, for example, those shedding earlier than average will also exhibit symptoms earlier than average. Specifically, we draw a single random value *U* from a uniform distribution on (0, 1) and calculate the latent and incubation periods as $F_L^{-1}(U)$ and $F_I^{-1}(U)$, where F_L and F_I are the cumulative distribution functions of the latent and incubation periods, respectively. Using the above parameters, we observe that shedding begins 0.5 to 1.2 days before symptom onset. However, shedding amounts do not ramp up to an appreciable level until around the time symptoms begin, as described next.

A daily shedding schedule (Table S2) was calculated in [26] by combining data from many published human exposure experiments. These data represent shedding amounts as function of time since exposure, rather than time since the first onset of shedding as required by our model. If we assume the exponentially distributed latent period described above is followed by a shedding schedule (for the fraction of total amount shed over time) that follows a lognormal distribution function (as also assumed in [28]), then the data in Table S2 would be described by:

$$A(t) = \int_0^t (1 - \mathrm{e}^{-r\tau})\, f(t - \tau; \mu, \sigma) \mathrm{d}\tau,$$

where *f* is the pdf of the lognormal distribution with parameters μ and σ. We fit this function to the above data set (where the values in the second column of Table S2 are $\log_{10}(A(t))$) and determined optimal parameter values μ = 0.2482 and σ = 0.3709.

Because shedding varies over time, the probability of an infectious person transmitting to a susceptible contact depends not only on the duration of contact, but also on when the contact started relative to the beginning of the infectious period. We use the following formula for the probability p_i that transmission occurs during a particular interaction:

$$p_i = 1 - \exp\{-k[F(t_i + d_i) - F(t_i)]\}$$

Here, *F* is the cumulative distribution function of the log-normally distributed shedding schedule defined above, t_i is the time of the start of the interaction in units of time since the infectious person's infectious period began, and d_i is the duration of the interaction. The parameter *k* can be interpreted as the product rh, where *h* is the total amount of virus shed by the infectious person over the entire infectious period, and *r* is the probability of infection per unit of virus shed by a contact in proximity, under an

exponential dose response model. Direct estimates of these values for cases in humans are scarce. However, we can calculate a nominal estimate for k using data from one commonly cited study [30] about an isolated set of influenza transmissions aboard an airliner. One initial case-patient had onset of symptoms 15 minutes after boarding, after which there was a 4.5 hour flight delay due to engine failure. During this 4.5 hour delay, 29 other passengers remained on board with the infectious index case-patient, and 25 (86%) of them subsequently became infected. As there was no mechanical ventilation in the cabin during this period and the passengers were reported to have been moving freely about the cabin, we consider the 29 passengers to have been in proximity with the index case-patient for 4.5 hours (d_i = 0.1875 days in the above equation). If we assume that the index case-patient experienced median-length latent (0.38 days) and incubation (1.4 days) periods, and given that onset of symptoms was 15 minutes into the 4.5 hour contact period, then we estimate that t_i in the above equation was 1.01 days, just prior to the peak of the shedding curve F. Then, applying p_i = 25/29 leads to a point estimate of k = 12. There were also 24 other passengers who did not stay on board during the entire 4.5 hour delay, and 13 (54%) were infected. The precise times that these other individuals were on board with the index case-patient are not known but were reported to be less than 3 hours. If the point estimate k = 12 is applied to our model, then the 54% attack rate would result from approximately 1.8 hours of exposure from the beginning of the flight delay.

We first applied the nominal value k = 12 in simulations for each school, and then applied double this value (k = 24) for purposes of testing the effects of simplifying network assumptions on the dynamics of larger outbreaks.

Given that a transmission will occur during a given interaction as described above, we generate a random time T that the transmission occurs using the following.

$$T = F^{-1}\left[F(t_i) - \frac{\ln(1 - Up_i)}{k}\right],$$

where U is a uniformly distributed random number on (0, 1). Again, T is measured in time since the infected person's infectious period began.

The formulas above are applied under the time-explicit, dynamic network version of our model, in which the precise timing of each person-to-person interaction is prescribed according to the WREN data. In simplified, static network versions in which interaction times are averaged (i.e., in which we assume that only the total duration of contact over one school day is known for each pair of people), a different formula is required. In this case, we make the simplifying assumption that interactions occur at a uniformly random rate throughout the school day. That is, if a pair of individuals has a total duration of contact d_{total} during a school day of length t_{school}, we assume that, at any moment of the school day, that pair is interacting with each other with probability $d_{\text{total}}/t_{\text{school}}$. Under this assumption, if one of the pair is infectious and the other susceptible, the expected probability p of transmission occurring between them is

$$p = 1 - \exp\left\{-k\left(\frac{d_{\text{total}}}{t_{\text{school}}}\right)[F(t_b) - F(t_a)]\right\}.$$

In this formula, t_a and t_b are the beginning and end times during which the infectious individual is at school, measured in time since the infectious period began. Given that a transmission will occur between a specific pair of individuals, we generate the random time T of transmission occurrence using

$$T = F^{-1}\left[F(t_a) - \frac{\ln(1 - Up)}{k\, d_{\text{total}}/t_{\text{school}}}\right].$$

In the homogeneous network versions of our model, we assigned the same duration to each edge in a given network or portion of a network, such that the resulting network produces the same average probability of transmission per edge as in the corresponding static, weighted network. Under each scenario, the homogeneous duration producing the correct average probability is a complicated function of the transmission parameter k and distributions of the timing of infection in relation to school attendance. We used our simulations on the static, weighted network to calculate what that average transmission probability per edge should be, and then ran further simulations to hone in on the correct homogeneous duration that produces the same average probability (Table 5).

Because the un-weighted, static contact network remained unchanged in the homogeneous and shuffled versions of our model, and we re-assigned the contact durations to produce the same average probability of transmission per edge, the corresponding R_0 also remained unchanged from version S2 (Table 4), as we defined it: the expected number of transmissions from an initially infected individual, given that every other individual is susceptible and every individual is equally likely to have the initial infection. The expected number of transmissions from a given, initially infected individual is the sum of the probabilities of transmission across all of that individual's contacts. Then, when averaging across all possible initially infected individuals, each edge in the network contributes exactly twice (once each from the individuals at either end of the edge) to the overall average R_0. Thus, as long as the average per-contact transmission probability is the same, changing the distribution of those probabilities does not affect R_0. However, the expected total outbreak size across all generations of transmission may differ substantially (Table 4), for example, if the new distribution changes the correlation between transmission probabilities and the level of clustering among contacts, which doesn't begin affecting outbreaks until the second generation of transmission.

S.1.5 Absenteeism behavior model – The symptoms status model was used to trigger potential school absenteeism behavior in the simulation. At the end of the incubation period when an individual moved into the symptomatic state, up to two random values were drawn to represent the timing of potential school absenteeism and subsequent return to school. First, a value was drawn for the number of days the individual will stay home. We assumed a mean of two days, based on observed data [3] and individual variation according to a Poisson distribution. This value does not necessarily correspond to the number of school days missed, as the number of days could include weekends. Under the Poisson distribution with mean of 2 days, there is approximately a 13.5% chance that the value is zero, or that the individual would not go or stay home from school at all. If that first value was greater than zero, a second random value was drawn to determine the delay between the onset of symptoms and leaving school. This delay was relevant only if symptoms onset happened to occur while or just before the individual was at school. For the second value, we drew a random delay from a uniform distribution with

limits of 0–2 hours. After this delay, the simulated individual entered a stay-at-home state in which the individual can no longer transmit infection to susceptible school contacts. The individual was kept in this state at least until the time of symptom onset plus the number-of-days-at-home parameter. If the end of this period fell during school hours, we extended the duration until the end of that school day. This time extension represented our assumption that individuals would not return to school in the middle of a school day after having been home sick.

Figure S1 – Serial intervals from transmission simulations

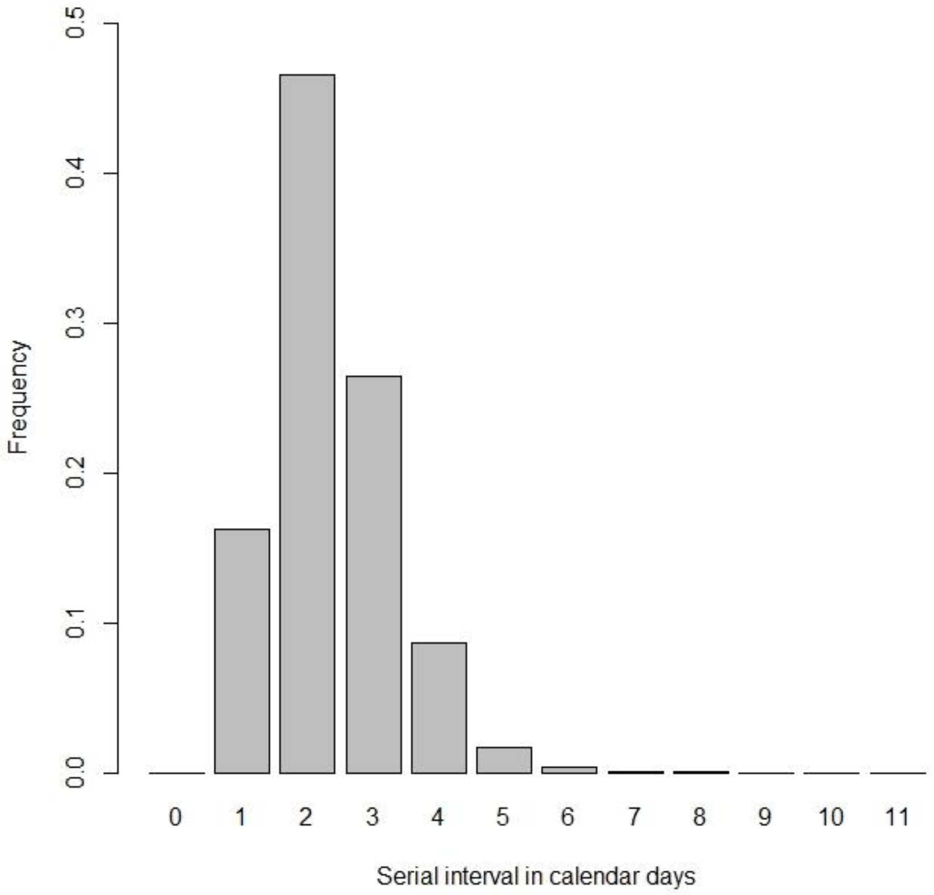

Results are from the Mid1 dynamic alternating network and higher transmissibility parameter. We defined the serial interval as the time between infection of a focus individual and the time of infection of his or her parent individual (the individual who transmitted infection to the focus individual). Initially infected individuals were excluded as parent individuals, as they could be infected outside of school hours. A 0-day serial interval means that the parent individual was infected earlier during the same school day that the focus individual was infected; these were extremely rare (5 occurrences out of more than 9.2 million transmissions). 54% of the 3-day intervals (14% of the total) and 76% of the 4-day intervals (7% of the total) occurred across a weekend.

Table S1. Human influenza data – first detection of shedding [26, 27]

Days after exposure	Shedding detected / Total
1	64 / 77
2	75 / 77
3	77 / 77

Table S2. Aggregated time-based human influenza shedding data [26]

Days after exposure	Log-10 viral concentration
1	1.9
2	3.0
3	2.6
4	2.2
5	1.5
6	1.1
7	0.7
8	0.3
9	0.3

Table S3: Student contact network measures using <1 meter contact distance criterion

	Mid1 Day 1	Mid1 Day 2	Mid1 Day 1&2	Elem1 Day 1	Elem1 Day 2	Elem1 Day 1&2
**Number of nodes**	590	590	590	339	339	339
**Mean degree (CV^2)**	51 (0.1)	52 (0.1)	83 (0.1)	27 (0.1)	36 (0.2)	46 (0.1)
**Mean strength (CV^2)**	98 (0.2)	99 (0.2)	98 (0.2)	86 (0.4)	121 (3.4)	103 (1.3)
**Mean edge duration (min/day)**	1.90	1.89	1.19	3.21	3.36	2.23
**Network density**	0.09	0.09	0.14	0.08	0.11	0.14
**Global clustering coefficient**	0.17	0.17	0.22	0.41	0.32	0.34
**Weighted clustering coefficient**	0.21	0.22	0.27	0.56	0.50	0.51
**Mean shortest path length**	1.98	1.98	1.86	2.50	2.13	1.99
**Mean most probable path length**	2.46	2.41	2.22	2.78	2.39	2.22
**Grade assortativity**	0.51	0.50	0.45	0.74	0.53	0.51
**Weighted grade assortativity**	0.74	0.72	0.73	0.95	0.92	0.93
**Class assortativity**				0.51	0.37	0.30
**Weighted class assortativity**				0.85	0.85	0.85

Elem1 = elementary school and Mid1 = middle school. Node degree = number of unique other students contacted for any duration, across one day or both days, by a given student; node strength = total duration (minutes per day) of contact across all contacts; edge duration = total duration (minutes per day) of contact for a given contact pair; network density = fraction of all possible node pairs that had a contact of any duration; global clustering = the probability that a connected triple is part of a triangle; weighted clustering as defined in [32]. Mean shortest path length is the average minimum number of edges (of any duration) needed to connect a random pair of nodes in the network. Mean most probable path length incorporates edge durations to calculate the average number of edges along the most probable pathway of transmission between any two nodes, under a sample transmission scenario (see Supplementary methods). Grade or class assortativity [33] quantifies the tendency for nodes in the same group to be connected to each other beyond (if positive) or below (if negative) what would be expected randomly. The weighted versions of assortativity use the total durations of within-group versus between-group edges rather than the number of edges.
